# Supplementary material for: Identification of photoperiod-induced specific miRNAs in the adrenal glands of Sunite sheep (Ovis aries)
Source: Front Vet Sci. 2022 Jul 22;9:888207. doi: 10.3389/fvets.2022.888207 (PMC9354845; doi:10.3389/fvets.2022.888207)
Supplement: Supplementary file 1 [file Data_Sheet_1.ZIP › Supplementary file/miRDeep2 file.zip!/miRDeep2 file/Know_Structure/SP42A2/oar-let-7d.pdf]

miRBase precursor : oar-let-7d
Total read count : 31202
oar-let-7d read count : 27893
remaining reads : 3309

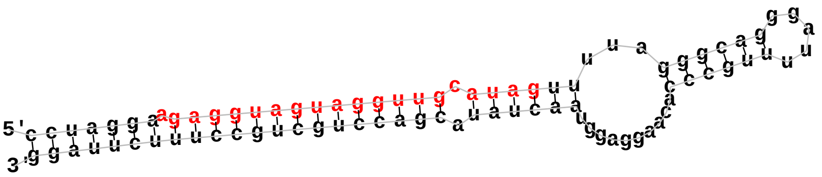

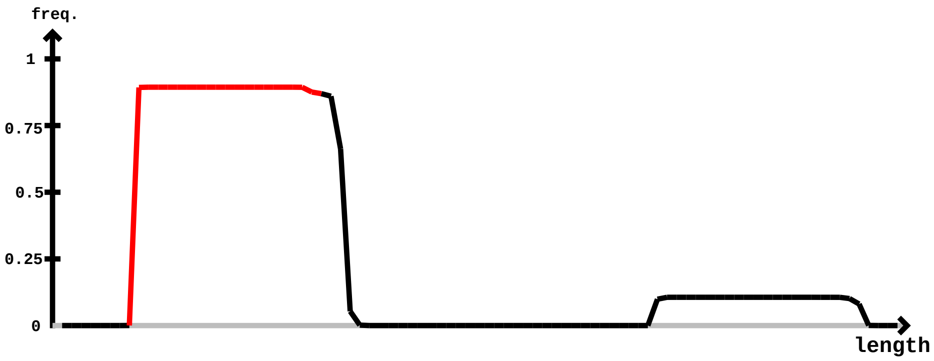


```
         oar-let-7d
5'- ccuaggaagagguaguagguugcauaguuuuagggcagggauuuugcccacaaggagguaacuauacgaccugcugccuuucuuagg   -3'    exp
    ((((((.((((((((((((((.((((((...((((((.....))))))..........)))))).))))))))))))))))))))     reads      mm         sample
    ......aagagguaguagguugcau..............................................................     1          0          seq
    ......aagagguaguagguugcauagu...........................................................     6          0          seq
    ......aagagguaguagguugcauaguu..........................................................     2          0          seq
    .......agagguaguagguugca...............................................................     15         0          seq
    .......agagguaguagguuAcau..............................................................     1          1          seq
    .......agagguaguagguugcau..............................................................     554        0          seq
    .......agagguaguagguuAcaua.............................................................     1          1          seq
    .......agagguaguagguugcaua.............................................................     185        0          seq
    .......agagguaguagguugcauag............................................................     300        0          seq
    .......agagguaguagguugcauagu...........................................................     6163       0          seq
    .......agagguaguagguugcauaguu..........................................................     18966      0          seq
    .......agagguaguagguugcauaguuu.........................................................     1614       0          seq
    .......agagguaguagguugcauaguuuu........................................................     51         0          seq
    .......agagguaguagguugcauaguuuua.......................................................     1          0          seq
    ........gagguaguagguugcau..............................................................     2          0          seq
    ........gagguaguagguugcauagu...........................................................     4          0          seq
    ........gagguaguagguugcauaguu..........................................................     23         0          seq
    ........gagguaguagguugcauaguuu.........................................................     1          0          seq
    .........agguaguagguugcauagu...........................................................     1          0          seq
    .........agguaguagguugcauaguu..........................................................     1          0          seq
    .........agguaguagguugcauaguuu.........................................................     1          0          seq
    ...........................................................aacuauacgaccugcugccuuucu....     1          0          seq
    .............................................................cuauacgaccugcugcc.........     1          0          seq
    .............................................................cuauacgaccugcugcU.........     1          1          seq
    .............................................................cuauacgaccugcugccu........     1          0          seq
    .............................................................cuauacgaccugcugccuu.......     3          0          seq
    .............................................................cuauacgaccugcugccuuu......     128        0          seq
    .............................................................cuauacgaccugcugccuuuc.....     587        0          seq
    .............................................................cuauacgaccugcugccuuucu....     2352       0          seq
    .............................................................cuauacgaccugcugccuuucuu...     12         0          seq
    ..............................................................uauacgaccugcugccuuu......     13         0          seq
    ..............................................................uauacgaccugcugccuuuc.....     51         0          seq
    ..............................................................uauacgaccugcugccuuucu....     153        0          seq
    ..............................................................uauacgaccugcugccuuucuu...     4          0          seq
    ...............................................................auacgaccugcugccuuuc.....     1          0          seq
```

oar-let-7d

```
ccuaggaagagguaguagguugcauaguuuuagggcagggauuuugcccacaaggagguaacuauacgaccugcugccuuucuuagg
...............................................................auacgaccugcugccuuucu....    1    0    seq
```
